# Understanding disparities in viral suppression among Black MSM living with HIV in Atlanta Georgia

**DOI:** 10.1002/jia2.25689

**Published:** 2021-04-06

**Authors:** Patrick S Sullivan, Justin Knox, Jeb Jones, Jennifer Taussig, Mariah Valentine Graves, Greg Millett, Nicole Luisi, Eric Hall, Travis H Sanchez, Carlos Del Rio, Colleen Kelley, Eli S Rosenberg, Jodie L Guest

**Affiliations:** ^1^ Department of Epidemiology Rollins School of Public Health Emory University Atlanta GA USA; ^2^ Department of Psychiatry Columbia University New York NY USA; ^3^ American Foundation for AIDS Research Washington DC USA; ^4^ Department of Medicine School of Medicine Emory University Atlanta GA USA; ^5^ Department of Epidemiology University of Albany Albany NY USA

**Keywords:** viral suppression, racial disparities, men who have sex with men, care cascade, HIV

## Abstract

**Introduction:**

Due to factors associated with structural racism, Black men who have sex with men (MSM) living with HIV are less likely to be virally suppressed compared to white MSM. Most of these data come from clinical cohorts and modifiable reasons for these racial disparities need to be defined in order to intervene on these inequities. Therefore, we examined factors associated with racial disparities in baseline viral suppression in a community‐based cohort of Black and white MSM living with HIV in Atlanta, GA.

**Methods:**

We conducted an observational cohort of Black and white MSM living with HIV infection in Atlanta. Enrolment occurred from June 2016 to June 2017 and men were followed for 24 months; laboratory and behavioural survey data were collected at 12 and 24 months after enrolment. Explanatory factors for racial disparities in viral suppression included sociodemographics and psychosocial variables. Poisson regression models with robust error variance were used to estimate prevalence ratios (PR) for Black/white differences in viral suppression. Factors that diminished the PR for race by ≥5% were considered to meaningfully attenuate the racial disparity and were included in a multivariable model.

**Results:**

Overall, 26% (104/398) of participants were not virally suppressed at baseline. Lack of viral suppression was significantly more prevalent among Black MSM (33%; 69/206) than white MSM (19%; 36/192) (crude Prevalence Ratio (PR) = 1.6; 95% CI: 1.1 to 2.5). The age‐adjusted Black/white PR was diminished by controlling for: ART coverage (12% decrease), housing stability (7%), higher income (6%) and marijuana use (6%). In a multivariable model, these factors cumulatively mitigated the PR for race by 21% (adjusted PR = 1.1 [95% CI: 0.8 to 1.6]).

**Conclusions:**

Relative to white MSM, Black MSM living with HIV in Atlanta were less likely to be virally suppressed. This disparity was explained by several factors, many of which should be targeted for structural, policy and individual‐level interventions to reduce racial disparities.

## 1 INTRODUCTION

Long‐standing structural racism – systems, institutions and processes that interact to produce and sustain inequities for racial and ethinc groups [1] – has led to stark racial disparities in the US HIV epidemic, with Black Americans experiencing higher rates of HIV infection than white Americans since early in the epidemic [2]. These racial disparities are also observed among men who have sex with men (MSM), who are >40 times more likely than other men to be living with HIV infection [3]. There has been considerable and important debate about what individual and societal factors give rise to Black/white disparities in HIV incidence [4, 5, 6, 7, 8, 9], but less has been written about racial differences in HIV treatment outcomes of people living with HIV (PLWH).

Studies show that racial disparities exist across the HIV treatment cascade [10] because of delayed access to and engagement in care [11], lower adherence to antiretroviral therapy (ARV) [12, 13, 14, 15], mistrust of providers [16, 17, 18] and stigma and discrimination [19, 20]. Lack of viral suppression results in increased morbidity and mortality among PLWH who are not suppressed, and contributes to onward HIV transmission [8, 21]. Thus, addressing racial disparities in viral suppression will improve health outcomes for Black MSM, and reduce the incidence of HIV in their sexual partners [22].

There are strong signals that the HIV care continuum endpoint, viral suppression, is less often achieved among Black MSM compared to white MSM, although nearly all data supporting this are from cross‐sectional analyses of clinical cohorts [23, 24]. Community‐recruited cohorts are critical because they less prone to selection biases towards men who are already in clinical care for HIV, like those recruited through clinical settings. Clinical studies may also have less detailed data about social determinants of health, which are critical determinants of successful clinical care and can form the basis for intervention development. In addition to retrospective analyses of clinical cohort data, prospective studies are needed to understand the causes of these disparities and to identify intervention targets to improve HIV care outcomes and reduce racial disparities.

An examination of racial disparities in care and prevention outcomes is timely because of the ongoing debate about the expansion of Medicaid in many Southern states [25], increased availability of private insurance under the Affordable Care Act [26], the national reckoning with racism and the Black Lives Matter movement [27] and the Ending the HIV Epidemic goals [28, 29]. Using baseline data from a prospective cohort of Black and white non‐Hispanic MSM living with HIV in Atlanta, GA, we studied levels of viral suppression and associated factors, focusing on modifiable factors that could be targeted to reduce racial disparities in viral suppression.

## 2 METHODS

### 2.1 Sampling, recruitment and enrolment

EngageMENt, a prospective cohort study, was designed to examine the factors that contribute to gaps and resiliency in HIV care and prevention between Black and white MSM in Atlanta, Georgia and define targets for intervention to reduce disparities [30]. To adequately describe HIV suppression and continuity of care among these groups, the study was designed to enrol equal numbers of Black and white MSM living with HIV infection (n = 200 each). Men were eligible for enrolment if they were previously diagnosed with HIV, or if they were diagnosed with HIV during screening for study eligibility. Participants were recruited in community venues and through advertisements in gay‐oriented magazines and on public transportation; participants were provided with incentives for participation: $60 for the completed baseline visit and the 12‐month visit, $75 for the completed 24‐month visit and $40 for each completed survey at months 3, 6 and 18. Data were collected from June 2016 to May 2017 for the baseline visits.

Self‐report of HIV‐positive status was confirmed during the baseline visit by HIV antibody testing. Additional eligibility criteria included male sex at birth and current male identity, self‐reported Black or white race, non‐Hispanic ethnicity, age ≥16 years, ability to complete study instruments in English, current residence in the Atlanta metropolitan statistical area, at least one male sex partner in the previous 12 months and willingness to provide at least two means of contact. Men were excluded if they were of Hispanic/Latino ethnicity, had plans to receive their HIV care outside of metro‐Atlanta in the next two years, or were currently enrolled in an HIV prevention or treatment clinical trial.

### 2.2 Biomedical measures

To assess plasma viral load, we used the Abbott RealTime HIV‐1 Assay, an in vitro reverse transcription‐polymerase chain reaction (RT‐PCR) assay for viral load measurements on the automated m2000 System from plasma (range of detection: 40 to 10,000,000 copies/mL) [31]. Lack of viral suppression was considered a viral load measurement of 40 copies/mL or greater, as measured by viral load testing at the baseline visit.

### 2.3 Explanatory variables

Our study was informed by Bronfenbrenner’s socio‐ecological model [32], as applied to HIV prevention by Baral *et al* [33]. As such, we conceptualized several possible explanatory factors for racial disparities in viral suppression, including Bronfenbrenner individual level (e.g. sociodemographics, access to treatment, psychosocial variables, behavioural characteristics and biological factors), exosystem level (insurance coverage, poverty) and macrosystem level (e.g. racism) [30]. Factors measured included: age, sexual identity, relationship status, educational attainment, income, employment, health insurance status, housing stability, incarceration in the past year (past year chosen over lifetime as it would more proximally relate to loss of medications during transition in or out of incarceration), time since HIV diagnosis, cigarette smoking, alcohol use, drug use and symptoms of depression and/or anxiety. Health insurance status was ascertained by two items, first: ‘Are you currently covered by health insurance (this includes Medicare or Medicaid)?” and the follow‐up item: “Do you use any of the following supplemental plans or assistance programmes?”’ with response options (all that apply): ADAP, Ryan White, Compassionate care programme, Free medication programme, Drug company programme, Health Insurance Continuation Programme. Participants were characterized as having: health insurance, Ryan White/ADAP/drug company programme, or none. Housing stability was ascertained by the item: ‘Which of these best describes your current housing situation?’ with response options: stable/permanent, transitional temporary and homeless. Depression and/or anxiety symptoms were determined by self‐report of diagnosis by a clinician and/or study assessment using the 4‐item Patient Health Questionnaire (PHQ‐4). Symptoms of anxiety and/or depression were ascertained as a combined score of 3 or more on the two depression items and/or a combined score of 3 or more on the two anxiety items) [34, 35].

To validate self‐reported heavy alcohol use (>1 drinks per day), the Emory Clinical Translational Research Laboratory assessed carbohydrate‐deficient transferrin (CDT) in blood specimens, a sensitive marker of recent heavy alcohol use (seven days) among both chronic and intermittent drinkers, using a solid phase‐phase sandwich enzyme linked immunosorbent assay. A CDT result of 2.6 or higher was interpreted as evidence of heavy drinking.[36] Qualitative screening for drugs was performed on urine for marijuana, methamphetamine, cocaine, phencyclidine (PCP), MDMA, barbiturates, benzodiazepines and methadone using a self‐contained, one‐step, 10‐drug panel test (iCup Drug Test Cup, BioScan Screening Systems). A positive urine screen or self‐reported use (past six months) were interpreted as having engaged in recent substance use.

Biological factors measured included current infection with Hepatitis C, Syphilis and Chlamydia and/or Gonorrhoea. We screened for antibodies to Hepatitis C in serum (Quest Diagnostics, Atlanta GA). Syphilis screening was conducted on serum using an FDA‐approved rapid plasma reagin (RPR) test with titres; positive RPR tests were confirmed with treponemal IgG test [37] The presence of C. trachomatis (CT) and N. gonorrhoea (NG) in self‐collected urethral and rectal swab specimens was determined using the Abbott Real Time CT/NG assay, an FDA‐cleared real‐time PCR assay for direct, qualitative detection of a region of the cryptic plasmid DNA of CT and the Opa gene of NG [31]. CD4 count was also assessed by Quest Diagnostics using flow cytometry.

### 2.4 Statistical analyses

We assessed the prevalence of viral suppression among participants at baseline, focusing on race‐stratified estimates of viral suppression. We descriptively summarized the above explanatory factors and compared the distribution of explanatory factors in Black and white MSM using χ2, Fisher’s exact and Wilcoxon tests. Then, we compared the explanatory variables in those without viral suppression to those with viral suppression with crude prevalence ratios (cPR), and exact 95% confidence intervals for each factor.

We next assessed which explanatory factors accounted for the racial disparity in lack of viral suppression. Using conditional margins logistic regression, we first estimated the age‐adjusted Black/white prevalence ratio (aaPR) for lack of viral suppression. Factors were then entered into the model one at a time; the extent to which they mediated the relationship between race and lack of viral suppression was evaluated by the change in the aaPR due to the addition of the covariate. Factors that attenuated the aaPR for race by ≥5%, were considered meaningful mediators [38]. All variables that met this criteria were then included in a multivariable model to assess how they collectively impacted the association between race and viral suppression. Statistical tests were two‐sided and *p* < 0.05 was considered statistically significant. SAS 9.4 was used for all statistical analyses.

## 3 RESULTS

A total of 400 participants were enrolled and completed a baseline visit (207 Black, 193 white). A total of 398 participants had data available for this analysis; of these, 52% were Black and 48% were white (Table 1). Black participants were younger, with 65% <40 years of age at baseline, whereas 67% of the white participants were ≥40 years of age. Mean age was 37 years for Black MSM and 44 years for white MSM. More Black (49%) than white (29%) participants were in the lower income bracket (<$20,000/year). More than 80% of participants had completed some college and nearly 70% were employed. Compared to all people living with HIV in Georgia, our sample had a lower proportion of Black participants (Atlanta: 73%) and an older age (median age in Atlanta: 24 to 44 years) [39].

**Table 1:** Explanatory sociodemographic variables by participant race among 398 Black and white MSM living with HIV enrolled in a cohort study, Atlanta, 2016 to 2017

	All participants (n = 398)		Black participants (n = 206)		White participants (n = 192)		*p*
	%	N	%	N	%	N	
Race							
White	48.2	192			100.0	192	
Black	51.8	206	100.0	206			
Age (years)							
18 to 24	7.5	30	70.0	21	30.0	9	<0.0001
25 to 29	11.6	46	69.6	32	30.4	14	
30 to 39	30.4	121	66.9	81	33.1	40	
40 to 49	24.6	98	41.8	41	58.2	57	
50+	25.9	103	30.1	31	69.9	72	
Sexual identity							
Homosexual/gay	91.5	364	48.4	176	51.6	188	<0.0001
Bisexual/other	8.5	34	88.2	30	11.8	4	
Relationship status							
Committed	29.6	117	47.9	56	52.1	61	0.298
Not	70.4	278	53.6	149	46.4	129	
Education							
Some college^a^	83.2	331	50.8	168	49.2	163	0.373
High school/GED or less	16.8	67	56.7	38	43.3	29	
Income, yearly^b^							
<$20,000	39.2	151	63.6	96	36.4	55	<0.0001
>$20,000	60.8	234	42.7	100	57.3	134	
Employed/student/disability							
Employed	69.1	275	49.1	135	50.9	140	0.437
Student	2.8	11	63.6	7	36.4	4	
Disability	13.1	52	57.7	30	42.3	22	
None	15.1	60	56.7	34	43.3	26	
Housing stability, current^c^							
Stable/permanent	76.2	301	47.8	144	52.2	157	0.007
Transitional/temporary/other	21.5	85	64.7	55	35.3	30	
Homeless	2.3	9	77.8	7	22.2	2	
Incarceration, previous 12 months							
Yes	11.1	44	68.2	30	31.8	14	0.021
No	88.9	354	49.7	176	50.3	178	

^a^ Associate’s degree and/or technical school, college, post graduate or professional school

^b^ 13 missing responses

^c^ 3 missing responses.

Over 86% of Black participants had prescription coverage for medicines to treat HIV, compared to 95% of white participants. Although most participants had private insurance (72%), there was a 19% difference in private insurance by race (62% Black, 81% white). The difference in private health insurance coverage was mitigated by a quarter of Black participants engaging in coverage with government programmes (Ryan White/ADAP) or pharmaceutical companies' drug assistance programmes. Both Black (49%) and white (67%) participants most commonly received HIV care in a doctor’s office. However, the second most common provider type differed by race: 22% of Black participants received care at the Health Department, and 16% of white participants received care at an AIDS Service Organization (data not shown in Table 1).

Differences were seen in housing instability by race. Although most (76%) participants reported stable/permanent housing, nearly a third of Black participants reported unstable housing, including homelessness, compared to about one in five white participants. Incarceration in the past year was twice as common among Black (15%) compared to white participants (7%). Although there was no statistically significant difference in depression or symptoms of anxiety by race, symptoms of these conditions were common among participants: about a third of all participants had a positive screen for depression or anxiety using the PHQ4 screener.

In terms of substance use, more than one in three participants currently smoked cigarettes. Problematic alcohol use (daily drinking or CDT > 2.6) was uncommon (7%), and did not differ by race. Multiple types of drug use varied by race. Marijuana was the most commonly used drug (50% overall; 62% among Black MSM, 37% among white MSM). White participants were more likely to have used methamphetamines than Black participants (23% vs. 12%). About a quarter of Black participants used cocaine compared to 16% of white participants, a difference that was not statistically significant.

Syphilis was the most common STI among participants; 34% of Black participants had a positive screening test, compared to 21% of white participants. Chlamydia/gonorrhoea were less commonly diagnosed and were not different by race.

### 3.1 Viral suppression

Of the 398 Black and white MSM who enrolled in the study, 294 (74%) were virally suppressed at baseline. Suppression differed by race: 67% of Black MSM and 79% of white MSM were virally suppressed, translating to a cPR of 1.8 (95% CI 1.2 to 2.5) (Table 2). Employment status, relationship status, binge drinking, cocaine use and hepatitis C status were not significantly associated with viral suppression.

**Table 2:** Explanatory behavioural and clinical variables by participant race among 398 Black and white MSM living with HIV enrolled in a cohort study, Atlanta, 2016 to 2017

	All participants (n = 398)		Black participants (n = 206)		White participants (n = 192)		*p*
	%	N	%	N	%	N	
Time since HIV diagnosis							
0 to 3 months	3.5	14	71.4	10	28.6	4	0.350
>3 months to 12 months	4.5	18	44.4	8	55.6	10	
>12 months to 5 years	20.4	81	56.8	46	43.2	35	
>5 years to 10 years	23.6	94	53.2	50	46.8	44	
>10 years	48.0	191	48.2	92	51.8	99	
Insurance coverage for ART							
Health insurance (current)	71.4	284	45.1	128	54.9	156	<0.001
ADAP/Ryan White/Drug company programme	19.1	76	65.8	50	34.2	26	
None	9.5	38	73.7	28	26.3	10	
Depression and/or anxiety							
Yes	32.7	130	56.9	74	43.1	56	0.141
No	67.3	267	49.1	131	50.9	136	
Smoking							
Current	36.4	145	53.1	77	46.9	68	0.017
Previously smoked (>100 cigarettes in life)	11.2	51	33.3	17	66.7	34	
Never	44.4	202	55.4	112	44.6	90	
Substance use, self‐report (past 6 months) or laboratory							
Alcohol heavy drinking	7.0	28	42.9	12	57.1	16	0.328
Marijuana	50.0	199	64.3	128	35.7	71	<0.0001
Methamphetamines	17.6	70	35.7	25	64.3	45	0.003
Cocaine	19.6	78	60.3	47	39.7	31	0.094
Hepatitis C^a^							
Positive	6.8	27	59.3	16	40.7	11	0.427
Negative	93.2	370	51.4	190	48.6	180	
Syphilis							
Reactive	27.6	110	63.6	70	36.4	40	0.003
Non‐reactive	72.4	288	47.2	136	52.8	152	
Chlamydia\gonorrhoea^b^							
Yes	13.0	51	60.8	31	39.2	20	0.162
No	87.0	340	50.3	171	49.7	169	

	IQR	Median	IQR	Median	IQR	Median	*p* ^c^
Viral load (copies/mL)	(20 to 48)	20	(20 to 288)	20	(20 to 20)	20	<0.0001
CD4 count (cells/μL)	(445 to 912)	670	(411 to 825)	602	(497 to 993)	702	<0.0001

^a^ 1 missing result

^b^ 7 missing results

^c^ Wilcoxon used to assess statistical significance.

Prevalence ratios were used to understand the extent to which the racial disparity can be accounted for by the explanatory factors that were measured (Table 3). All prevalence ratios were in the hypothesized direction, with less viral suppression in participants in the following groups: younger age, lower income levels, unstably housed or homeless, no insurance coverage for ART prescriptions, positive screening for symptoms of depression/anxiety, currently smoking cigarettes, reported marijuana use, reported methamphetamines use, and those diagnosed with STIs at the baseline visit. Length of time since HIV diagnosis was associated with viral suppression levels: those who were recently diagnosed with HIV were less likely to be virally suppressed compared to those who were diagnosed with HIV more than 10 years ago.

**Table 3:** Explanatory sociodemographic, behavioural and clinical variables by viral suppression and crude associations with viral suppression among 398 Black and white MSM living with HIV enrolled in a cohort study, Atlanta, 2016 to 2017

	Virally suppressed (n = 294)		Not virally suppressed (n = 104)		*p*	Prevalence ratio	95% CI
	%	N	%	N			
Race							
White	81.3	156	18.8	36	0.001	Reference	
Black	67.0	138	33.0	68		1.76	(1.24 to 2.51)
Age (years)							
18 to 24	63.3	19	36.7	11	0.000	3.15	(1.54 to 6.42)
25 to 29	58.7	27	41.3	19		3.55	(1.88 to 6.70)
30 to 39	68.6	83	31.4	38		2.70	(1.49 to 4.89)
40 to 49	75.5	74	24.5	24		2.10	(1.11 to 3.98)
50+	88.3	91	11.7	12		Reference	
Sexual identity							
Homosexual/gay	73.9	269	26.1	95	0.962	Reference	
Bisexual/other	75.0	21	25.0	7		1.01	(0.56 to 1.83)
Relationship status							
Committed	73.5	86	26.5	31	0.902	1.02	(0.71 to 1.47)
Not	74.1	206	25.9	72		Reference	
Education							
Some college^a^	77.0	255	23.0	76	0.001	Reference	
High school/GED or less	58.2	39	41.8	28		1.82	(1.29 to 2.57)
Income, yearly^b^							
<$20,000	64.9	98	35.1	53	0.001	1.75	(1.25 to 2.45)
>$20,000	79.9	187	20.1	47		Reference	
Employed/student/disability							
Employed	74.9	206	25.1	69	0.713	Reference	
Student	63.6	7	36.4	4		1.45	(0.64 to 3.26)
Disability	69.2	36	30.8	16		1.23	(0.78 to 1.94)
None	75.0	45	25.0	15		1.00	(0.61 to 1.62)
Housing stability, current^c^							
Stable/permanent	78.4	236	21.6	65	<0.0001	Reference	
Transitional/temporary/other	62.4	53	37.6	32		1.74	(1.23 to 2.47)
Homeless	22.2	2	77.8	7		3.60	(2.39 to 5.44)
Incarceration, previous 12 months							
Yes	61.4	27	38.6	17	0.045	1.57	(1.04 to 2.38)
No	75.4	267	24.6	87		Reference	
Time since HIV diagnosis							
0 to 3 months	28.6	4	71.4	10	0.001	3.59	(2.32 to 5.57)
>3 months to 12 months	66.7	12	33.3	6		1.68	(0.82 to 3.43)
>12 months to 5 years	69.1	56	30.9	25		1.55	(1.00 to 2.40)
>5 years to 10 years	73.4	69	26.6	25		1.34	(0.86 to 2.08)
> 10 years	80.1	153	19.9	38		Reference	
Insurance coverage for ART							
Health insurance (current)	80.3	228	19.7	56	<0.0001	Reference	
ADAP/ryan white/drug company programme	71.1	54	28.9	22		1.47	(0.96 to 2.25)
None	31.6	12	68.4	26		3.47	(2.52 to 4.78)
Depression and/or anxiety							
Yes	63.8	83	36.2	47	0.002	1.69	(1.22 to 2.35)
No	78.7	210	21.3	57		Reference	
Smoking							
Current	63.4	92	36.6	53	0.001	1.68	(1.19 to 2.36)
Previously smoked (>100 cigarettes in life)	86.3	44	13.7	7		0.63	(0.30 to 1.32)
Never	78.2	158	21.8	44		Reference	
Substance use, self‐report (past 6 months) or laboratory							
Alcohol heavy drinking	82.1	23	17.9	5	0.301	0.67	(0.30 to 1.50)
Marijuana	66.8	133	33.2	66	0.001	1.74	(1.23 to 2.46)
Methamphetamines	57.1	40	42.9	30	0.001	1.90	(1.35 to 2.66)
Cocaine	67.9	53	32.1	25	0.184	1.30	(0.89 to 1.89)
Hepatitis C^d^							
Positive	63.0	17	37.0	10	0.173	1.47	(0.87 to 2.49)
Negative	74.9	277	25.1	93		Reference	
Syphilis							
Reactive	63.6	70	36.4	40	0.004	1.64	(1.18 to 2.28)
Non‐reactive	77.8	224	22.2	64		Reference	
Chlamydia\gonorrhoea^e^							
Yes	60.8	31	39.2	20	0.022	1.63	(1.10 to 2.41)
No	75.9	258	24.1	82		Reference	

^a^ Associate’s degree and/or technical school, college, post graduate or professional school

^b^ 13 missing responses

^c^ 3 missing responses

^d^ 1 missing result

^e^ 7 missing results

**Figure 1:**
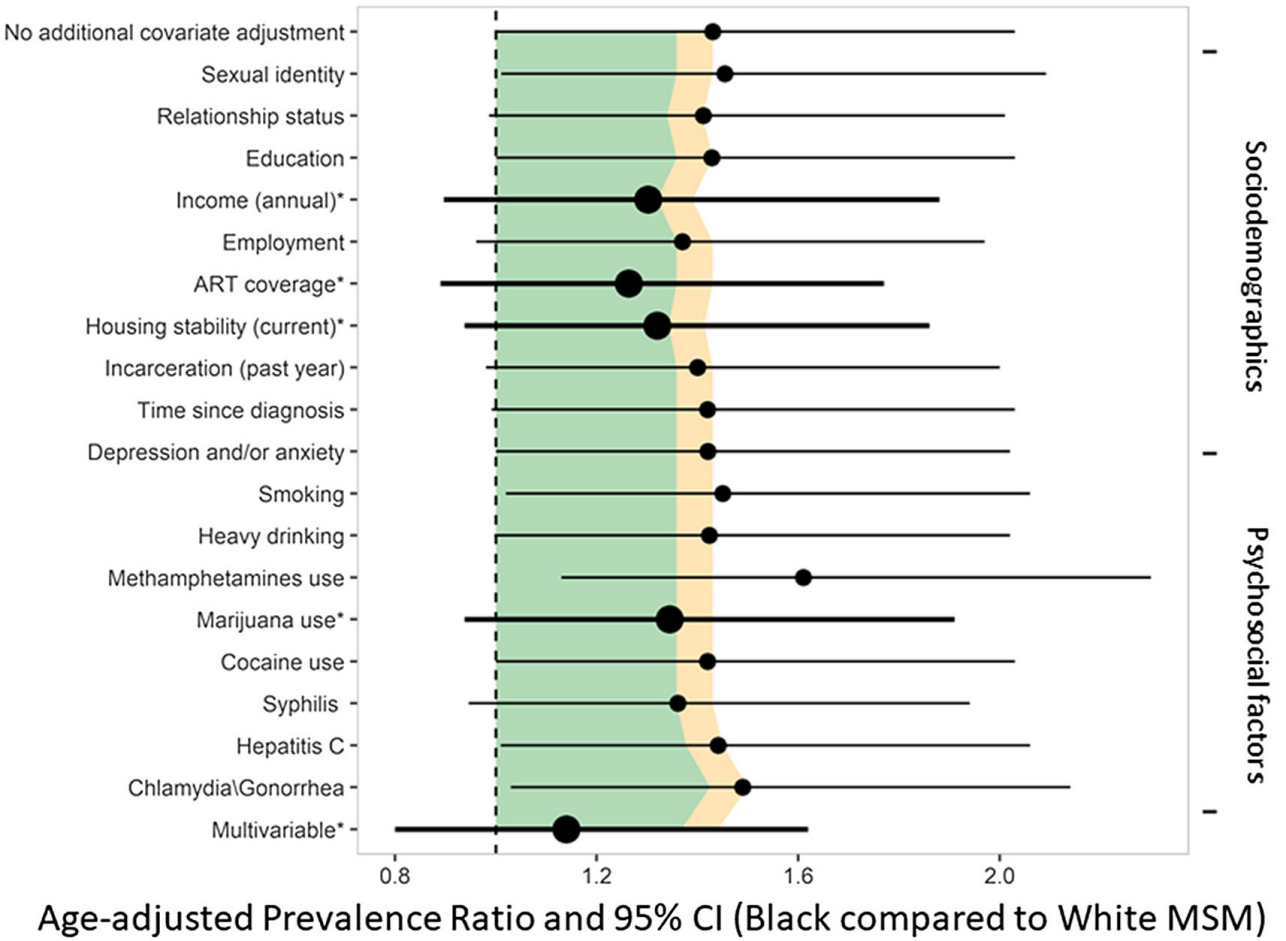
Age‐adjusted Black‐White Prevalence ratios for viral suppression from multivariable models in a community‐based sample of 398 Black and White MSM living with HIV, Atlanta, 2016 to 2017. **(a)** Yellow region indicates covariate‐adjusted PR for race that are between 0% and 5% less than the PR for race with just age adjustment, whereas green region indicates covariate‐adjusted PR that are more than 5% less, indicating meaningful attenuation of the race disparity in lack of viral suppression. **(b)** Multivariable adjustment included the four variables that meaningfully attenuated the race disparity in lack of viral suppression (income, ART coverage, housing stability, marijuana use), which are indicated with asterisks after their labels.

Because younger age was positively associated with lack of viral suppression and the age structures of the white and Black participants were different (with Black MSM being younger, on average), we calculated an age‐adjusted prevalence ratio (aaPR) as the reference value for analyses of attenuation of racial disparity by explanatory factors. The aaPR for lack of viral suppression among Black MSM vs. white MSM was 1.4 (95% CI 1.0 to 2.0). The aaPR for lack of viral suppression by race was then examined adjusting for one explanatory variable at a time to determine if these additional variables meaningfully attenuated the relationship between race and viral suppression. (Figure 1) Four modifiable factors reduced the race aaPR by 5% or more: annual income, current housing stability, ART coverage and marijuana use. When all four of these variables that individually and meaningfully attenuated the age‐adjusted racial disparity were included in a multivariable model, the aaPR for the association between race and viral suppression fell to 1.1 (95% CI 0.8 to 1.6). The combination of these four variables accounted for a 21% reduction in the difference seen in viral suppression rates by race.

## 4 DISCUSSION

Viral suppression is the cornerstone of improving clinical outcomes for people living with HIV, and people living with HIV who are virally suppressed are effectively unable to transmit HIV to their sex partners [40, 41, 42]. Yet, in the United States, Black men in care for HIV are less likely to be prescribed ART, are more likely to report side effects from ART, are more likely to have intentionally stopped ART for 2 days or more, and are less likely to have viral suppression [43]. A CDC analysis examining men in care for HIV reported that while 89% of Black men were taking ART, only 52% were virally suppressed [43]. We found that 67% of Black MSM living in the community with HIV were virally suppressed – a significantly lower proportion than for white MSM. In 2017, the Georgia Department of Public Health reported, based on surveillance data, that 55% of all Black Georgians living with HIV were virally suppressed, compared to 72% of white Georgians [44]. Although our overall finding of lower viral suppression in Black MSM was similar to the surveillance study, our analysis provided additional information by focusing on Black MSM (vs. all Black Georgians) and by identifying the factors that explained the disparity. Our analysis was a snapshot of viral suppression; other studies have suggested that continuous suppression is even worse for Black MSM [45].

There were four social‐environmental factors that meaningfully attenuated the racial disparity and, collectively, they accounted for statistical difference in viral suppression between Black and white MSM: lack of access to ART coverage, being unstably housed, having lower income, and marijuana use. Having a source of payment for ART medications should be understood as residing in Bronfenbrenner’s [32] and Baral’s [33] public policy levels. Although the AIDS Drug Assistance Program (ADAP) can support some aspects of HIV care, periodic requirements for recertification can lead to lapses in care. Additionally, Georgia has not yet acted to expand Medicaid coverage [46]. Expansion of Medicaid has been shown to be associated with improved outcomes for other health conditions [47], and the sharp decline in new HIV diagnoses in Massachusetts has been partially attributed to Medicaid expansion [48]. Similarly, states with Medicaid expansion have had better uptake of PrEP among key high‐risk populations for HIV infection [49]. States also have an option for more targeted Medicaid waivers that have been used to enable access to HIV care [50]. Georgia has proposed two Medicaid waivers related to HIV [51], but these proposals have been criticized as insufficient measures to close the gap in access to care for people living with HIV in Georgia [52]. In short, improved payment coverage for antiretroviral therapies and medical care are imminently modifiable risks for lack of viral suppression. Lack of prescription coverage and health insurance disproportionately affected Black MSM in our study and were strong drivers of the inequity in viral suppression that we observed. We call on the State of Georgia to expand Medicaid coverage broadly; the Metropolitan Atlanta HIV Health Services Planning Council estimates that 75% of Georgians living with HIV would be covered under Medicaid with full Medicaid expansion – compared to the 17% currently covered (personal communication – Jeff Graham, Georgia Equality).

Housing instability was common, disproportionately affecting Black MSM, and was positively associated with not being virally suppressed. Multiple other studies have shown housing instability to be common among PLWH and to predict worse HIV treatment outcomes [53, 54, 55, 56, 57]. Our finding that housing instability partially accounts for racial disparities in HIV treatment outcomes is a novel finding, but not a surprising one. Housing instability has been identified as a critical risk for the health of people living with HIV for decades [58], has been associated with poor healthcare access [59], has been associated with other poor health outcomes [54, 60] and has been recently associated with higher viral load in a clinical cohort of young Black MSM in care for HIV infection [25]. Housing instability is a result of a complex interplay between individual vulnerabilities and broader structural factors. However, multiple randomized controlled trials have found providing housing assistance to independently improve outcomes among PLWH who experience housing instability [61, 62], and thus it is a modifiable contextual factor [54, 63]. To increase access to housing services for PLWH, the Health Resources and Services Administration recommends building new partnerships between public and private stakeholders, funding innovative strategies to address housing needs in PLWH, and involving PLWH with housing needs in this process [64]. Our findings suggest that these efforts also need to be more effectively directed to the needs of Black MSM.

The prevalence of marijuana use that we observed (50%) was towards the higher end of the range that has been reported among other studies of PLWH (14% to 60%). Major differences from previous studies are that we also used an objective biomarker to assess recent use, whereas the recall times of the other studies varied and all but one [65] of them were among clinical cohorts [66, 67, 68, 69, 70, 71]. Marijuana use was positively associated with not being virally suppressed. There have been conflicting findings in studies on the impact of marijuana use and HIV treatment outcomes. Some studies report a negative association between marijuana use and ART adherence [72], and others report no effect [69, 73]. One study reported no association overall between marijuana use and ART adherence, but found a positive association among those who experienced nausea (and thus they might have been using marijuana for medical reasons) [74]. Regarding marijuana use and viral load, one study reported a negative association [75] and two others found no effect [70, 73]. Our data on the association of marijuana use and lack of viral suppression in the context of explaining racial disparities in HIV treatment outcomes is novel, especially as use was assessed using an objective biomarker [76]. Our prior analyses of MSM in Atlanta have documented differential misclassification of self‐reported drug use by race [76]. Because these current analyses are cross‐sectional, we cannot ascertain the direction of the relationship. It is possible that the impairment caused by marijuana use on memory, planning and organizational skills [77, 78, 79] negatively impacts ART adherence and hence viral suppression. In contrast, individuals who are not virally suppressed may be more likely to use marijuana because of its therapeutic effects. [80, 81] This is an area that merits further exploration using longitudinal data.

Regarding stimulants, the prevalence of cocaine and methamphetamine use that we observed (20% and 18% respectively) was slightly higher than what was observed among MSM living with HIV in the 2017 National HIV Behavioral Surveillance (NHBS) survey (18% and 12% respectively). [82] Again, our study was unique in that we also used an objective biomarker to assess recent use, which is important given under‐reporting of stimulant use [76]. We found methamphetamine use, but not cocaine use, to be positively associated with not being virally suppressed. Methamphetamine use has been associated with worse HIV treatment outcomes across multiple studies [83, 84, 85, 86, 87, 88]. Methamphetamine use was more prevalent among white participants than Black participants as has typically been reported [89, 90], although this may be changing [91]. Therefore, methamphetamine use was the only variable that, when controlled for, meaningfully strengthened the estimate of racial disparity in viral suppression. Given the high levels of methamphetamine use that we observed in the study, with substantial methamphetamine use among Black MSM (12%) and reports that methamphetamine use is rising [30] and increasingly linked to overdose deaths [92], this is an important area for future surveillance.

Younger participants were less likely to be virally suppressed than older participants, and Black MSM were younger, on average, than white MSM. This is consistent with other research showing that young Black MSM, in particular, experience suboptimal outcomes across the HIV care continuum [24, 93, 94, 95, 96, 97, 98]. This is particularly concerning as rates of HIV diagnoses have been increasing among young Black MSM [99, 100, 101]. Over the past decade, young Black MSM have experienced increases in new HIV infections; from 2006 to 2009, new infections increased 48% in young Black MSM (ages 13 to 29) [99], and from 2011 to 2015, new infections increased 30% in young Black MSM (ages 25 to 34) [100]. By 2016, Black MSM accounted for 25% of all new HIV diagnoses, more than half of which were among young Black MSM (ages 13 to 34) [100]. We controlled for age to ensure that we were fairly assessing modifiable factors associated with lack of viral suppression, but in this cross‐sectional analysis we might not have exhaustively captured all possible pathways between exposures and outcomes (e.g. incarceration leading to housing instability and unemployment, which could impact health insurance and viral suppression. In programmes, the disproportionate impact of HIV in younger Black MSM should speak to the need for age‐appropriate and culturally tailored programmes for HIV prevention and all aspects of the care cascade including treatment.

Our data are subject to important limitations. Our data are baseline data from a cohort study, and directionality of association cannot be inferred from the cross‐sectional analysis. We also followed the participants over time, and time‐to‐event analyses will provide more direct evidence of which exposures might be temporally associated with incident loss of viral suppression. Second, our sample is subject to selection bias. Although we recruited men through multiple approaches and attempted to integrate approaches to minimize this bias (e.g. venue time space sampling), we were more likely to recruit men who attended venues popular with gay men and who responded to gay‐themed online advertisements. This sampling bias might have introduced selection bias across dimensions of socioeconomic status and care outcomes. We also might have had differential recruitment rates by race. Third, our exposures were subject to misclassification because most were self‐reported. Of those exposures that meaningfully explained lack of viral suppression, housing status, income and insurance status relied solely on self‐report. Our viral suppression outcome and substance use were measured objectively in the study and were not subject to social desirability bias [102].

## 5 CONCLUSIONS

Health inequities arise from societal inequities, and structural racism is at the core of policies that perpetuate them [1, 103]. In Atlanta, disparities in viral suppression for Black MSM arise from lack of equitable access to medical care and stable housing. Because Black MSM experience lower rates of viral suppression, they also experience worse clinical outcomes, and their partners experience higher risks of acquiring HIV. Based on the cross‐sectional baseline data from our study, expansion of Medicaid and improving access to stable housing for people living with HIV are critical steps towards reducing and, eventually, eliminating these inequitable outcomes for Black MSM living with HIV.

There is still more to understand about the mechanisms of achieving and sustaining viral suppression for Black and white MSM. Similar prospective analyses are needed to describe the factors associated with losing viral suppression to document whether rates of loss of viral suppression are also higher for Black MSM living with HIV in this cohort who have achieved suppression, and to identify precedents to loss of suppression, has been reported in other cohorts. Understanding precedents of loss of viral suppression will inform the development of interventions to reduce loss of suppression in the future. Today, we have an obligation to Black MSM living with HIV to address the equitable availability of medical care and stable housing in Georgia. Policy interventions will have substantial positive effects towards decreasing racial inequities in HIV outcomes.

## ETHICS STATEMENT

This study was reviewed and approved by the Institutional Review Board of Emory University on March 5, 2016. Informed consent was administered in person at the study clinic, before the first enrolment visit.

## COMPETING INTERESTS

The authors Sullivan, Knox, Jones, Taussig, Valentine‐Graves, Millett, Luisi, Hall, Sanchez, Del Rio, Kelly, Rosenberg and Guest report no competing interests.

## AUTHORS’ CONTRIBUTIONS

PS, GM, TS, CK, ER and CDR designed the research. PS, JT, MVG, NL, TS, CK, ER and JG conducted study procedures. PS, JK, JJ, NK, EH, ER and JG analysed the data. PS, JK and JG wrote the initial draft of the manuscript. PS, JK, JJ, JT, MVG, GM, NL, EH, TS, CDR, CK, ER and JG provided critical input to the manuscript draft. The final manuscript draft was approved by PS, JK, JJ, JT, MVG, GM, NL, EH, TS, CDR, CK, ER and JG.

## ABBREVIATIONS

aaPR, age‐adjusted Prevalence Ratio; ART, Antiretroviral therapy; HIV, Human Immunodeficiency Virus; MSM, Men who have sex with men; PLWH, People living with HIV; PR, Prevalence Ratio; US, United States.

## FUNDING

This work was supported by the National Institutes of Health (R01AI112723 and K01AA028199). This work was supported by the Center for AIDS Research at Emory University (P30AI050409).
